# Host Searching and Aggregation Activity of Recently Fed and Unfed Bed Bugs (*Cimex lectularius* L.)

**DOI:** 10.3390/insects2020186

**Published:** 2011-05-04

**Authors:** Matthew D. Reis, Dini M. Miller

**Affiliations:** Department of Entomology, Virginia Tech, 216A Price Hall, Blacksburg, VA 24061, USA; E-Mail: matthewreis174@gmail.com

**Keywords:** bed bug, *Cimex lectularius*, movement, aggregation activity

## Abstract

Groups of starved, virgin adult male or female bed bugs were stimulated to search for a host by the presence of a heated artificial feeder. Some of the bed bug groups were allowed to obtain a blood meal and some were not. After the removal of the feeder, bed bugs were observed throughout the scotophase to record their searching and aggregation behavior. Groups of male and female bed bugs that were unable to obtain a blood meal continued to search in the arena for the majority of the scotophase. Bed bugs that were able to obtain a blood meal returned to their shelter to aggregate 30 min after feeding. Overall, the proportion of bed bugs aggregating in shelters during the scotophase was significantly greater for those that had fed successfully than those that had not. However, all bed bugs, regardless of feeding status, began to return to shelters to aggregate 2 h prior to the photophase.

## 1. Introduction

The bed bug, *Cimex lectularius* L., has five immature life stages, each requiring a blood meal in order to develop into the next instar. Adult bed bugs also must take blood meals throughout their lives in order to reproduce [1]. Although *C. lectularius* can feed on other animals, it has a long history of feeding almost exclusively on human hosts.

Hungry bed bugs are stimulated to feed when they detect an increase in ambient CO_2_. When a human enters an infested room and lies down to rest, the ambient CO_2_ in the room begins to increase. This increase in CO_2_ stimulates the bed bug to leave the harborage and begin searching. When the bed bug is near the host, the host's body heat serves as an additional directional cue. Once in contact with the host, the bed bug begins to feed. After feeding to repletion, the bed bug will leave the host's body and presumably return to a harborage to aggregate with other bed bugs [2].

Bed bugs are typically nocturnal, feeding during the scotophase when the host is asleep. However, this nocturnal behavior may be a relatively recent evolutionary development. Usinger [2] suggested that the ancestors of *C. lectularius* fed on cave-dwelling bats. When humans moved into the caves and either lived with (doubtfully) or removed (more likely) the bats, the bed bugs had to change their habits to cope with the new, diurnal host. Romero *et al.* [3] suggested that the bed bug's nocturnal feeding activity is a facultative change in response to the hosts' circadian rhythm. The bed bugs' ability to make this facultative change would indicate that their endogenous clock may be somewhat flexible with regard to environmental conditions and feeding status.

The feeding status of another blood feeding hemipteran, *Triatoma infestans* Klug, has been known to influence its circadian activity. When a regular host is present, *T. infestans* exhibits unimodal searching activity, leaving its shelter at the beginning of the scotophase, feeding, and returning to the harborage prior to photophase [4]. However, in the absence of a host, searching activity was found to be bimodal or crepuscular, where the insect was actively searching (moving) for multiple hours at both dusk and dawn [5]. It was also found that if *T. infestans* was subjected to periods of starvation, the length of both searching periods increased. Interestingly, because these studies were conducted to evaluate *T. infestans* host searching behavior, very little of their behavior *after* feeding was described.

We would expect the host searching behavior of the *C. lectularius* to be similar to that of *T. infestans* due to the fact that both insects live in close proximity to the diurnal host, and thus have nocturnal feeding behaviors. Romero *et al.* [3] produced the most recent and comprehensive study regarding the bed bugs' circadian rhythm and locomotor activity. Similar to the *T. infestans*, Romero *et al.* [3] found that bed bugs starved for one week were more active (*i.e.*, displayed more nocturnal movement) than those that had fed two days prior to testing.

The Romero *et al.* [3] study is an example of how recent research has focused on bed bug movement and searching behavior. However, very little is known about what bed bugs do immediately after feeding, when they are no longer searching for a host. Bed bugs feed every 5–7 days. Therefore, the majority of the population on any given day is not searching or feeding. While it is well known that bed bugs, fed and unfed, hide in cracks and crevices during the photophase, we do not know how long after feeding (or not feeding) that they begin this aggregation behavior.

The purpose of this study was to document the scotophase and aggregation activity of two groups of bed bugs; those that were stimulated to leave an established harborage and feed, and those who were stimulated to leave the harborage but were unable to feed. These activity patterns were documented for virgin adult male and female bed bugs.

## 2. Materials and Methods

### 2.1. Origin and Maintenance of Bed Bugs

Bed bugs (*C. lectularius*) were collected in 2008 from a hotel in downtown Cincinnati, Ohio and maintained at the Dodson Urban Pest Management Laboratory in Blacksburg, Virginia. The bed bugs were reared in plastic jars covered at one end with a cloth mesh. Two pieces of cardboard were placed inside the jars to allow bed bugs to crawl up and stick their mouth parts through the cloth mesh to feed. Bed bugs were fed once a week on chicken blood formulated with sodium citrate as an anti-coagulant. An artificial feeder using circulating hot water maintained the chicken blood at 35.5 °C. Between feedings, the jars with bed bugs were stored in an environmental chamber at 23–26 °C, 68.9% RH, and photoperiod of 12:12 L:D.

### 2.2. Preparation of Bed Bugs

Virgin male and female bed bugs were obtained by removing numerous fifth-instar bed bugs from rearing containers and placing them into glass vials. Each fifth-instar bed bug was fed and allowed to molt to adulthood. Adult males and females were separated as soon as they molted from the fifth instar. In each experiment, 10 bed bugs of the same sex were fed and placed on a 2 × 2 cm shelter made of filter paper folded once (Whatman® #1, Maidstone, England). These bed bugs were then left in their respective shelters to starve for 2–3 weeks.

### 2.3. Bioassay Arena

Square plastic display boxes (23 × 23 × 2 cm with a lid; Nunc™, Rochester, NY) were used as bioassay arenas. The bottoms of the arenas were covered with brown Kraft paper (40#, Westrick Paper Company, Jacksonville, FL) and the sides were coated with fluon (AGC Chemicals Americas, Inc., Exton, PA) to prevent bed bug escape. The lid of each arena had a circular hole (6 cm) cut in the center, which was covered in cloth mesh. Bioassay arenas were held in a room maintained at 26–27 °C, 20–40% RH, and a 12L:12D photoperiod (lights on from 08:00 to 20:00). After the bed bugs had been starved, a single shelter containing 10 bed bugs was placed inside each arena, approximately 4 cm away from the center. A total of five arenas (50 bed bugs) were used for each treatment combination (fed and unfed males, fed and unfed females; 200 bed bugs total).

### 2.4. Bioassay Design

**Figure 1:**
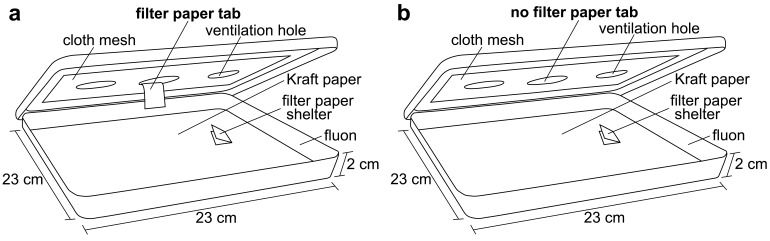
Bioassay arenas used to document bed bug scotophase activity. Bed bugs were located inside the filter paper shelter at the right: (**a**) An arena where bed bugs were able to climb up a paper tab to reach an artificial feeder; (**b**) An arena where paper tab was absent so bed bugs could detect the heat of the feeder but not reach it. Illustration: WR Kuhn.

Bed bugs are known to be stimulated by host cues, including heat [6]. To stimulate the bed bug searching behavior, an artificial feeder containing heated chicken blood was placed directly on the cloth mesh in the center of the arena lid at 21:00. The bed bugs would then leave their shelter to search for the heat source. Half of the arenas (five replicates) were provided with a filter paper tab hanging from the bottom of the cloth mesh. The bed bugs were able to climb up the tab and access a blood meal from the feeder (Figure 1a). These bed bugs were given 30 min to feed to repletion and drop off of the tab. The second half of the arenas (five replicates) were not provided with a paper tab so the bed bugs would be stimulated, but not able to obtain a blood meal from the artificial feeder (Figure 1b). The bed bugs without the paper tab were allowed to forage (unsuccessfully) for 30 min. After this time, the artificial feeder was removed and a digital video camera (Sony DCR-TRV250 Digital Video Recorder, Sony) was placed over each arena to record all bed bug behavior. The bed bugs' behavior was captured from 21:00 to 11:00.

### 2.5. Statistical Analysis

After removal of the artificial feeder, the number of bed bugs in each paper shelter was recorded (via camera) at 30 min intervals for 12 h. The proportion of bed bugs aggregating in the paper shelter was calculated at each interval. These proportions were transformed using the arcsine transformation [7] for analysis. The proportion of bed bugs in each shelter recorded between 21:30 and 6:00 was compared using repeated measures analysis of variance (ANOVA; SAS® 9.1, SAS Institute Inc., Cary, NC) [8]. Values of *P* ≤ 0.05 were used to indicate significance. Means were separated using Tukey-Kramer HSD [9]. Proportions of bed bugs in shelters recorded 2 h prior to the photophase were not included in the statistical analysis. Aggregation behavior prior to the photophase was not included in the analysis because it was determined to be influenced by the circadian rhythm to which the bed bugs had been habituated in the laboratory for two years.

## 3. Results

Prior to application of the artificial feeder (20:00–21:00), approximately 65% of all test bed bugs were located inside their shelters. After the artificial feeder was placed on the bioassay arena (21:00), test bed bugs were immediately observed leaving the shelter to forage. In bioassays where bed bugs were allowed to contact the artificial feeder, 96% of the bed bugs fed successfully.

**Figure 2:**
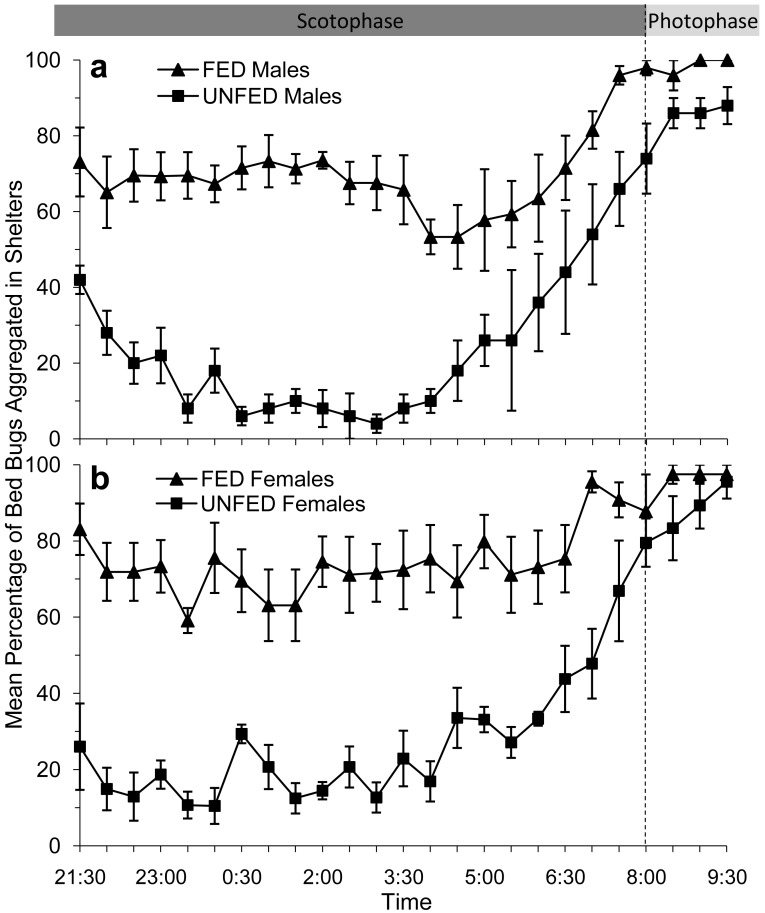
Mean percent of fed and unfed male (**a**) and female (**b**) bed bugs (± SE) inside shelters after being stimulated to forage by an artificial feeder for 30 min at 21:00 (n = 5). Figure shows untransformed data.

In bioassays where male bed bugs were provided access to the artificial feeder (fed), ∼70% of the males returned to the paper shelter to aggregate within 30 min after feeding. More than 50% of the fed males remained inside the shelter for the duration of the scotophase (Figure 2a). Males that had been stimulated to forage but were not provided access to the artificial feeder (unfed) behaved very differently from the fed males. Immediately after the feeder was removed, ∼40% of the male bed bugs returned to the shelter, however, they did not remain there. For a considerable portion of the scotophase (23:00–05:00) less than 80% of the unfed male bed bugs were inside the shelter. Instead, unfed male bed bugs were observed moving around the arena until 06:00, after which time they began returning to the shelter.

Similar to the male bed bugs, the majority (∼80%) of female bed bugs that were allowed to take a blood meal from the artificial feeder (fed) also returned immediately to the shelter to aggregate (Figure 2b). Female bed bugs that did not have access to the feeder (unfed) continued to move around the arena for 6–8 h after the feeder had been removed (Figure 2b). In fact, only 10–35% of the female bed bugs were recorded in the shelter prior to 06:00.

From approximately 04:00 to 06:00, or 2 h prior to the onset of the photophase, we observed that the remaining fed and unfed bed bugs of both sexes began to return to the shelter to aggregate. This trend was so consistent for each of the test groups that we had to conclude that the circadian rhythm to which the bed bug had been habituated had a greater influence on their foraging/aggregation behavior than their feeding status. In fact, by the time the photophase began at 08:00, over 70% of the bed bugs in each test group were inside the shelter.

The mean percentages of bed bugs in each test group aggregating inside a shelter between 21:30 and 06:00 are shown in Table 1. Overall, ∼70% of fed bed bugs, both male and female, were aggregating in shelters throughout a majority of the test period. Conversely, ∼80% of unfed bed bugs were searching the arena.

The repeated measures ANOVA indicated that there was a significant difference in the proportion of fed and unfed bed bugs aggregating in shelters throughout the test period (21:30–06:00; Table 2). The mean adjusted proportion of fed males aggregating in shelters was significantly greater than that of unfed males (Table 2). Similarly, the mean adjusted proportion of fed females aggregating in shelters was significantly greater than that of unfed females. There was no significant interaction between the sex of the bed bug (male or female) and their aggregation behavior when their feeding status was the same (fed or unfed). There were significant differences between males and females when their feeding status was different (one fed; the other unfed).

## 4. Discussion

The bioassays in this study document bed bug behavior immediately after feeding and contrast those behaviors with bed bugs that have been stimulated to feed, but were not able to obtain a blood meal. We observed that starved bed bugs, upon being stimulated to feed, left their shelter and searched for a host. If they were able to feed, they returned back to a shelter almost immediately (∼80% in 30 min) and remained there for the duration of the scotophase. However, if the bed bugs did not obtain a blood meal, they continued to search for a host for many hours. Interestingly, almost all bed bugs (fed or unfed) began returning to the shelter to aggregate at approximately 2 h prior to the photophase. At the start of the photophase (08:00), more than 80% of all bed bugs (fed or unfed) were aggregating in the harborage.

Bed bugs that were unable to obtain a blood meal continued to search (for hours) throughout the arena for a host. Lehane [10] described host location as a three-step process: (1) appetitive searching that is driven by hunger; (2) activation, where oriented behavior is exhibited; and (3) attraction, where host stimuli bring the insect into contact with the host. The bed bugs in this study were in the appetitive stage. The presence of the artificial feeder triggered the searching behavior, but the bed bugs' inability to reach the feeder once stimulated resulted in their continuing to search throughout the night until approximately 06:00.

The searching behavior we observed in the unfed bed bug bioassays was similar to Mellanby's [11] observations in the field. Mellanby [11] observed that bed bugs foraged all night, but that the majority of bed bug foraging activity took place between the hours of 03:00 and 05:30. Our results indicated that unfed bed bugs were very active for a longer period of time, with the majority of foraging activity occurring between 23:30 and 06:00. There may be several reasons why our results differ from those reported by Mellanby [11]. One is most likely that Mellanby made his observations in the field. Our bioassays were conducted in the laboratory with colonies that had been acclimated to laboratory conditions for two years. In addition, the feeding status of the bed bugs in the Mellanby [11] study was not specified, and most likely unknown.

There would appear to be two reasons why fed male and female bed bugs would rapidly return to a shelter to aggregate after feeding. The first reason is to find a mate. We have observed bed bugs, particularly males, attempting to mate with conspecifics immediately after feeding. In this study, none of the bed bugs were provided with potential mates. However, the fed males were observed attempting to mount each other upon returning to the harborage before settling down for the remainder of the (disappointing) scotophase. The second reason that fed bed bugs would rapidly seek shelter is that they are very vulnerable to being crushed after feeding. They must therefore leave the host and retreat to safety [12].

While the fed bed bugs' bloated condition may stimulate them to leave the host, we still know very little about how they select a harborage. The presence of feces and exuvia in a harborage stimulate aggregation behavior in the laboratory [13], however, observations of bed bug aggregation in the field suggest that there is more to harborage selection than simply the presence of feces and exuvia. In the field we have observed bed bugs aggregating in many locations within the host's sleeping room. A significant number of these aggregations have been found at distances between 10–15 m from where the host sleeps. When recording the make-up of these aggregations, we observed all different life stages aggregating together. Some of these individuals had obviously fed during the previous scotophase, as they were still engorged and bright red with the blood of the host. These observations suggested that fed bed bugs may travel a significant distance to a shelter after feeding. One of our early hypotheses was that bed bugs exhibited some degree of harborage loyalty and would return to that harborage after feeding even when closer harborages were available. This hypothesis was supported by field observations of established harborages with numerous fecal stains located at more than 5–10 m from where the host slept. However, laboratory assays designed to assess harborage loyalty, (e.g., where bed bugs inside two established shelters were placed inside the same arena and fed to see if they would return to their shelter of origin after feeding) typically resulted in bed bugs from different shelters all aggregating together in a single shelter after feeding [14].

So while the results of this study suggest that bed bugs return to their harborage immediately after feeding, our field observations indicate that bed bug aggregation behavior may be influenced by additional factors. More study is needed to elucidate all of the factors that influence fed bed bug aggregation behavior, including the initial stimulus to leave the host, the distance that a bed bug will travel to a shelter, and bed bug harborage selection.

## 5. Conclusions

Our study contributes new data to the body of knowledge on the subject of bed bug host searching and aggregation behavior. Specifically, bed bug behavior immediately after feeding was documented. Bed bugs that were stimulated to forage but were unable to obtain a blood meal continued to search throughout the majority of the scotophase, only returning to the shelter to aggregate 2 h prior to the photophase. In contrast, bed bugs that were stimulated to forage and were able to feed successfully, typically returned to the shelter within 30 min to aggregate. Those few that did not return to the shelter immediately also began to return to the shelter 2 h prior to the photophase. While the results of this study were very consistent and logical, additional study is needed to explain the more complicated aggregation behaviors that are often observed in the field.

## Figures and Tables

**Table 1:** Mean percentage of male and female bed bugs (*n* = 5) aggregating (every 30 min from 21:30–06:00 throughout the test period) in shelters after being stimulated to feed.

**Sex of adult bed bug (50 individuals each treatment)**	**Feeding status after artificial feeder was removed**	**Mean percentage of bed bugs in shelters (±SE)**
Male	Fed	66.2 ±3.7
Male	Unfed	16.9 ±3.9
Female	Fed	71.6 ±6.6
Female	Unfed	20.6 ±1.7

**Table 2:** Comparison of treatment interactions for male and female bed bugs (*n* = 5) aggregating in shelters (checked every 30 min from 21:30–06:00) after being stimulated to feed.

**Sex**	**Feeding status**	**Sex**	**Feeding status**	***t*-value**	Adjusted *P*-value*
Female	Fed	Female	Unfed	9.39	<0.0001
Female	Fed	Male	Fed	1.21	0.63
Female	Fed	Male	Unfed	10.11	<0.0001
Female	Unfed	Male	Fed	-8.18	<0.0001
Female	Unfed	Male	Unfed	0.72	0.89
Male	Fed	Male	Unfed	8.90	<0.0001

*Mean adjusted proportions of bed bugs aggregating in shelters were compared using repeated measures ANOVA. Means separated using Tukey-Kramer HSD (df = 16; α ≤ 0.05).
